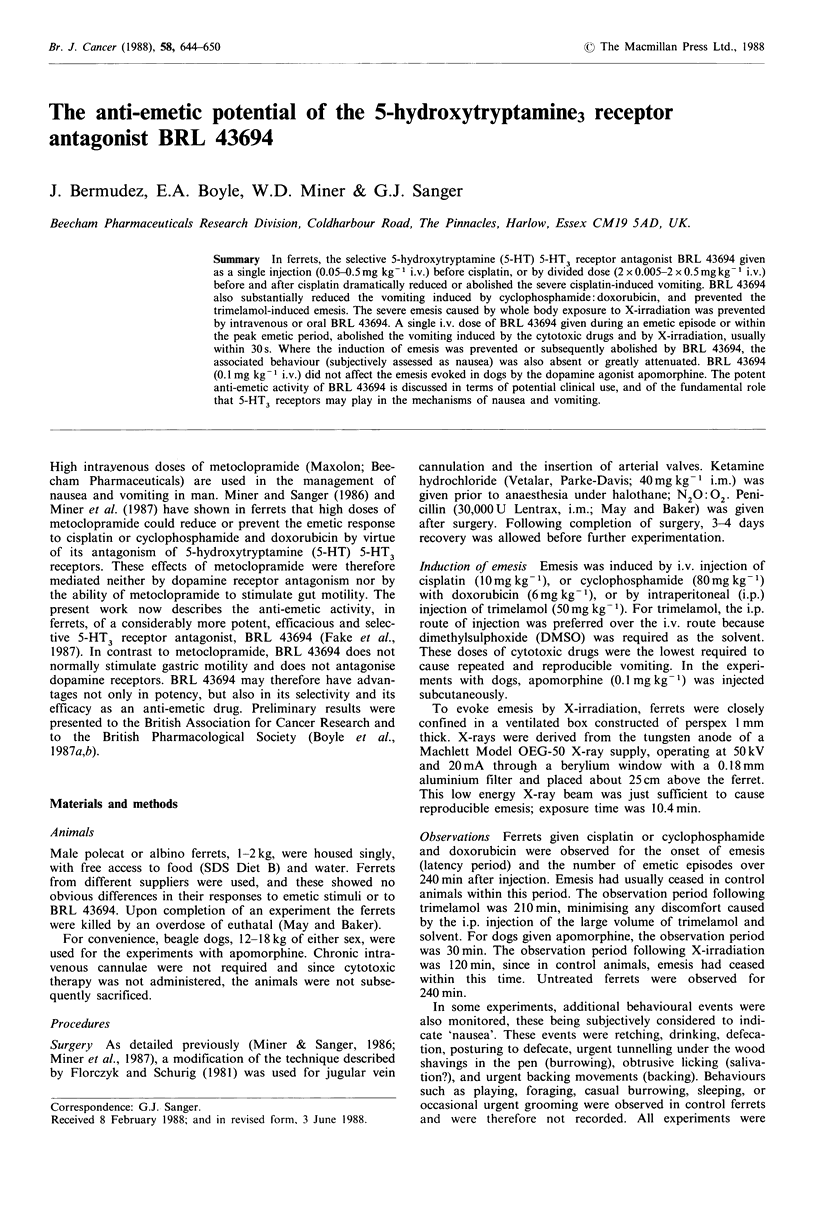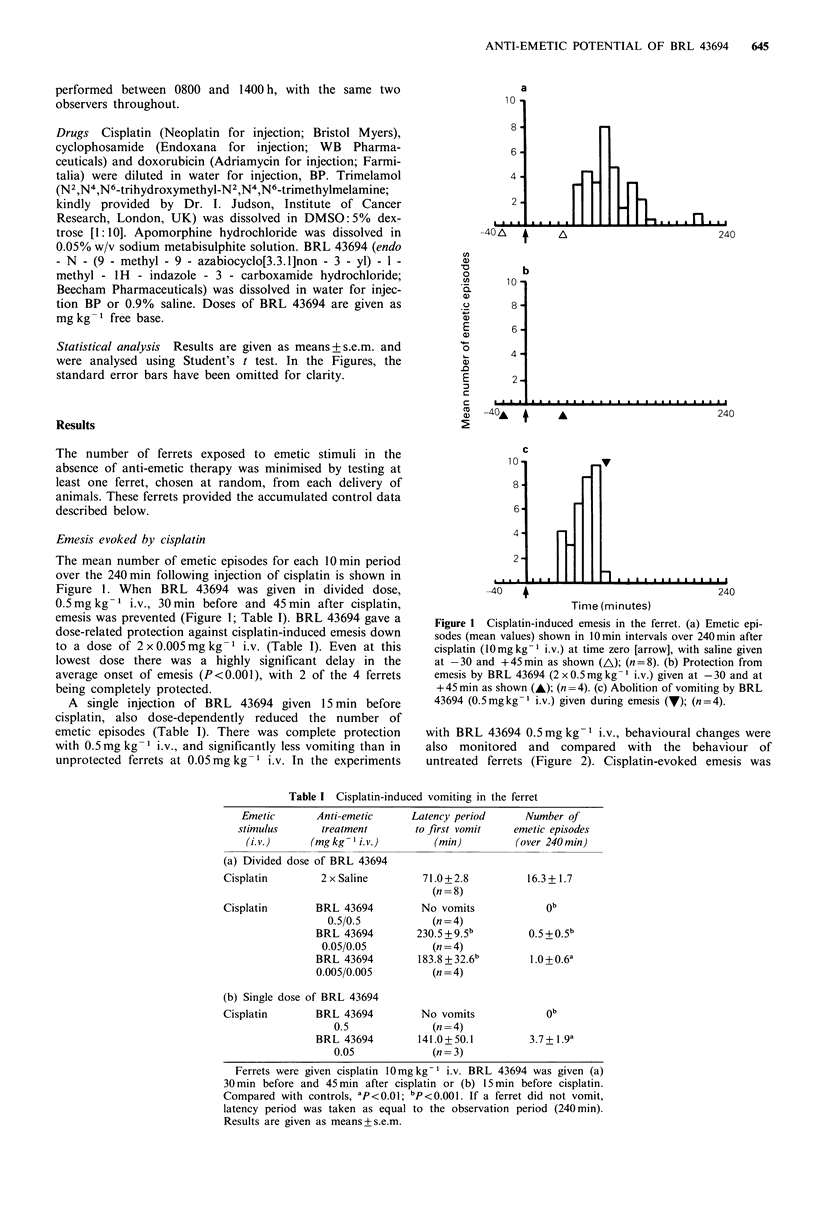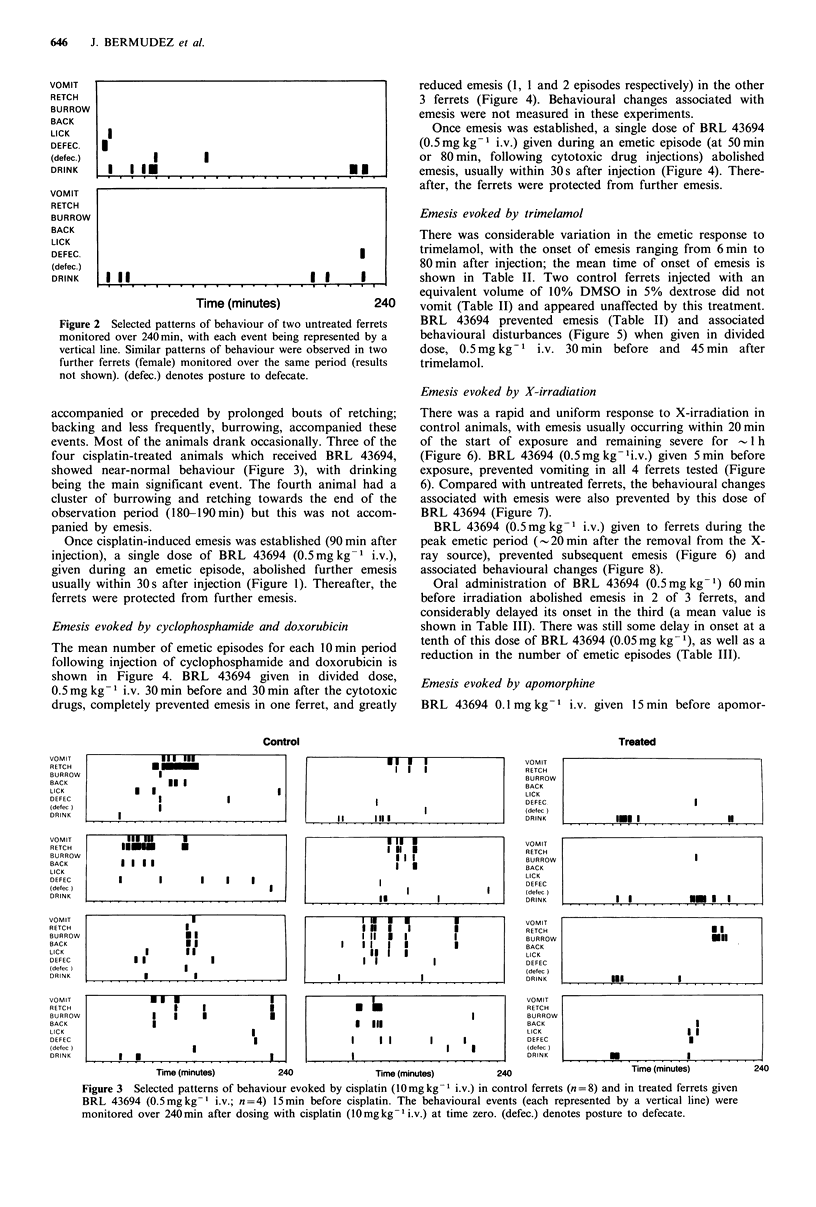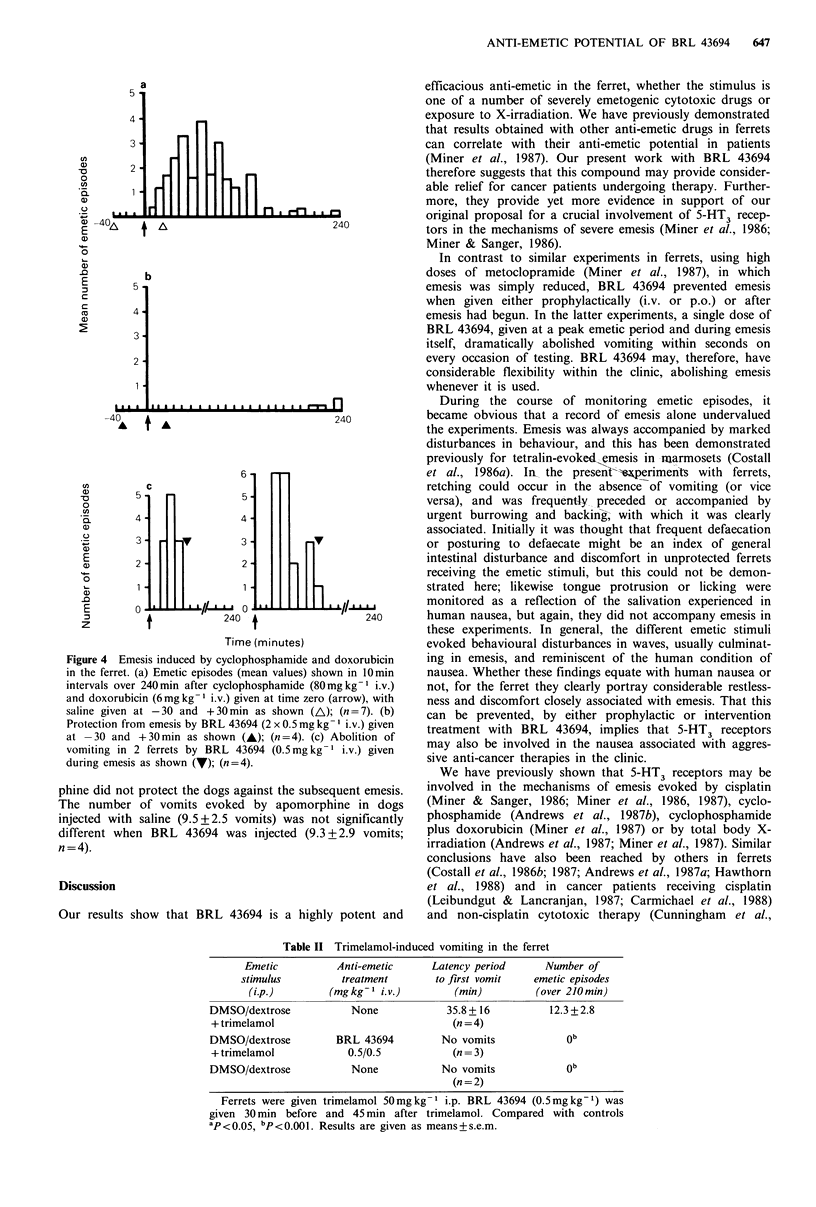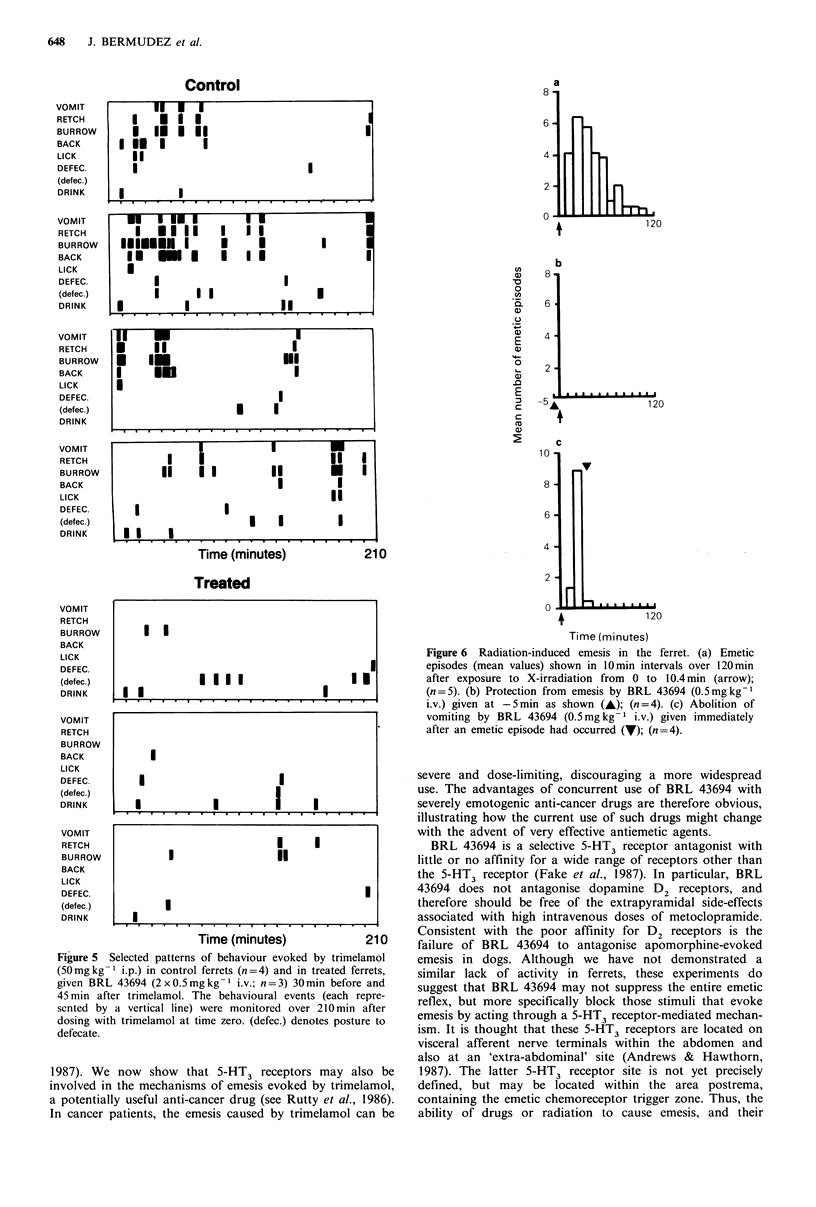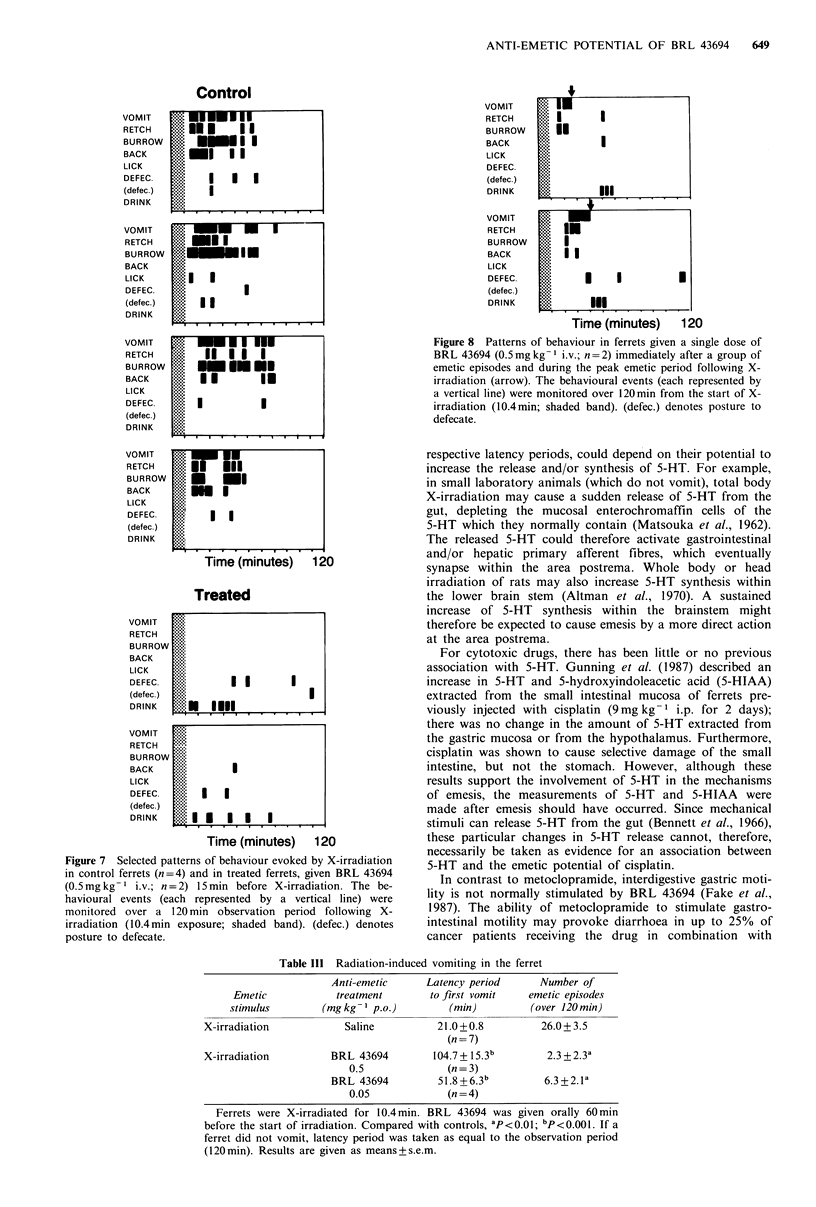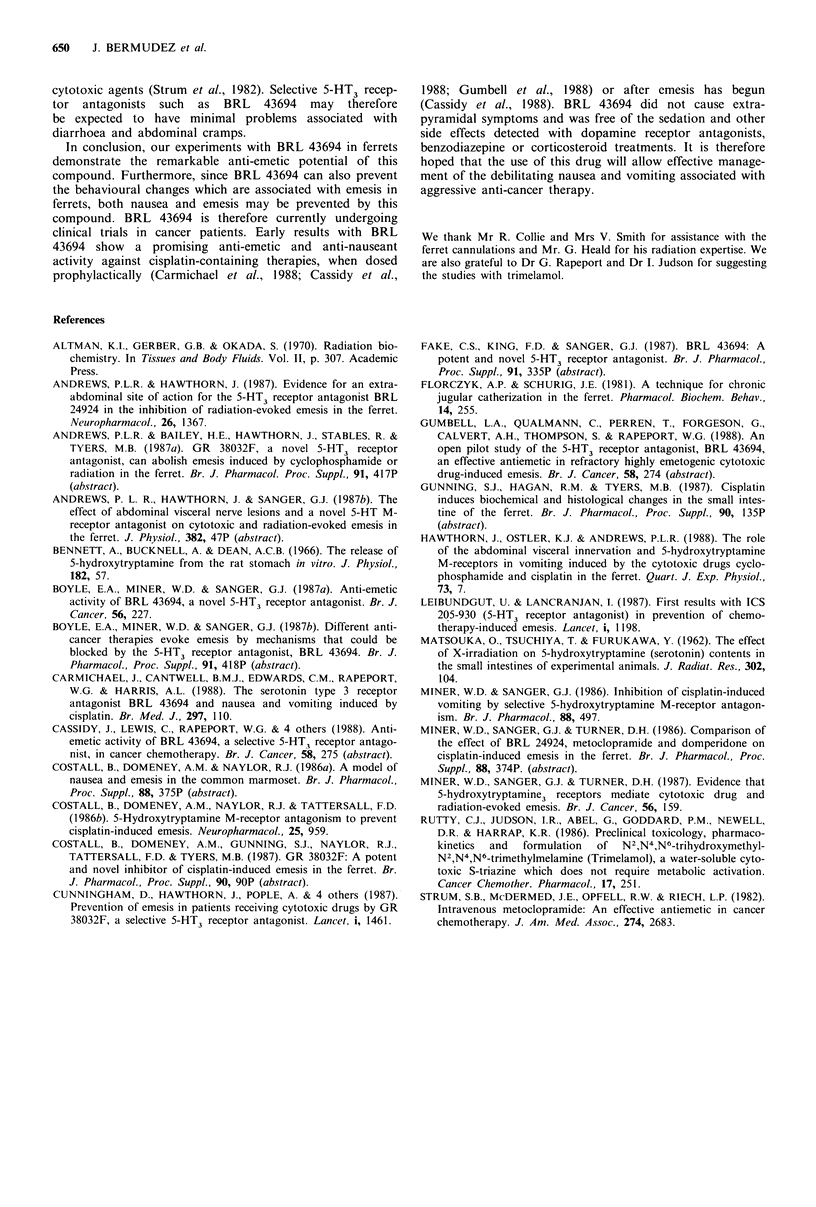# The anti-emetic potential of the 5-hydroxytryptamine3 receptor antagonist BRL 43694.

**DOI:** 10.1038/bjc.1988.277

**Published:** 1988-11

**Authors:** J. Bermudez, E. A. Boyle, W. D. Miner, G. J. Sanger

**Affiliations:** Beecham Pharmaceuticals Research Division, Harlow, Essex, UK.

## Abstract

In ferrets, the selective 5-hydroxytryptamine (5-HT) 5-HT3 receptor antagonist BRL 43694 given as a single injection (0.05-0.5 mg kg-1 i.v.) before cisplatin, or by divided dose (2 x 0.005-2 x 0.5 mg kg-1 i.v.) before and after cisplatin dramatically reduced or abolished the severe cisplatin-induced vomiting. BRL 43694 also substantially reduced the vomiting induced by cyclophosphamide:doxorubicin, and prevented the trimelamol-induced emesis. The severe emesis caused by whole body exposure to X-irradiation was prevented by intravenous or oral BRL 43694. A single i.v. dose of BRL 43694 given during an emetic episode or within the peak emetic period, abolished the vomiting induced by the cytotoxic drugs and by X-irradiation, usually within 30 s. Where the induction of emesis was prevented or subsequently abolished by BRL 43694, the associated behaviour (subjectively assessed as nausea) was also absent or greatly attenuated. BRL 43694 (0.1 mg kg-1 i.v.) did not affect the emesis evoked in dogs by the dopamine agonist apomorphine. The potent anti-emetic activity of BRL 43694 is discussed in terms of potential clinical use, and of the fundamental role that 5-HT3 receptors may play in the mechanisms of nausea and vomiting.


*Br. J. Cancer* (1988), **58**, 644–650 © The Macmillan Press Ltd., 1988

### The anti-emetic potential of the 5-hydroxytryptamine$_3$ receptor antagonist BRL 43694

J. Bermudez, E.A. Boyle, W.D. Miner & G.J. Sanger

*Beecham Pharmaceuticals Research Division, Coldharbour Road, The Pinnacles, Harlow, Essex CM19 5AD, UK.*

**Summary** In ferrets, the selective 5-hydroxytryptamine (5-HT) 5-HT$_3$ receptor antagonist BRL 43694 given as a single injection (0.05–0.5 mg kg$^{-1}$ i.v.) before cisplatin, or by divided dose (2×0.005–2×0.5 mg kg$^{-1}$ i.v.) before and after cisplatin dramatically reduced or abolished the severe cisplatin-induced vomiting. BRL 43694 also substantially reduced the vomiting induced by cyclophosphamide: doxorubicin, and prevented the trimelamol-induced emesis. The severe emesis caused by whole body exposure to X-irradiation was prevented by intravenous or oral BRL 43694. A single i.v. dose of BRL 43694 given during an emetic episode or within the peak emetic period, abolished the vomiting induced by the cytotoxic drugs and by X-irradiation, usually within 30 s. Where the induction of emesis was prevented or subsequently abolished by BRL 43694, the associated behaviour (subjectively assessed as nausea) was also absent or greatly attenuated. BRL 43694 (0.1 mg kg$^{-1}$ i.v.) did not affect the emesis evoked in dogs by the dopamine agonist apomorphine. The potent anti-emetic activity of BRL 43694 is discussed in terms of potential clinical use, and of the fundamental role that 5-HT$_3$ receptors may play in the mechanisms of nausea and vomiting.

High intravenous doses of metoclopramide (Maxolon; Beecham Pharmaceuticals) are used in the management of nausea and vomiting in man. Miner and Sanger (1986) and Miner *et al.* (1987) have shown in ferrets that high doses of metoclopramide could reduce or prevent the emetic response to cisplatin or cyclophosphamide and doxorubicin by virtue of its antagonism of 5-hydroxytryptamine (5-HT) 5-HT$_3$ receptors. These effects of metoclopramide were therefore mediated neither by dopamine receptor antagonism nor by the ability of metoclopramide to stimulate gut motility. The present work now describes the anti-emetic activity, in ferrets, of a considerably more potent, efficacious and selective 5-HT$_3$ receptor antagonist, BRL 43694 (Fake *et al.*, 1987). In contrast to metoclopramide, BRL 43694 does not normally stimulate gastric motility and does not antagonise dopamine receptors. BRL 43694 may therefore have advantages not only in potency, but also in its selectivity and its efficacy as an anti-emetic drug. Preliminary results were presented to the British Association for Cancer Research and to the British Pharmacological Society (Boyle *et al.*, 1987*a*,*b*).

### Materials and methods

### *Animals*

Male polecat or albino ferrets, 1–2 kg, were housed singly, with free access to food (SDS Diet B) and water. Ferrets from different suppliers were used, and these showed no obvious differences in their responses to emetic stimuli or to BRL 43694. Upon completion of an experiment the ferrets were killed by an overdose of euthatal (May and Baker).

For convenience, beagle dogs, 12–18 kg of either sex, were used for the experiments with apomorphine. Chronic intravenous cannulae were not required and since cytotoxic therapy was not administered, the animals were not subsequently sacrificed.

### *Procedures*

*Surgery* As detailed previously (Miner & Sanger, 1986; Miner *et al.*, 1987), a modification of the technique described by Florczyk and Schurig (1981) was used for jugular vein cannulation and the insertion of arterial valves. Ketamine hydrochloride (Vetalar, Parke-Davis; 40 mg kg$^{-1}$ i.m.) was given prior to anaesthesia under halothane; $N_2O:O_2$. Penicillin (30,000 U Lentrax, i.m.; May and Baker) was given after surgery. Following completion of surgery, 3–4 days recovery was allowed before further experimentation.

*Induction of emesis* Emesis was induced by i.v. injection of cisplatin (10 mg kg$^{-1}$), or cyclophosphamide (80 mg kg$^{-1}$) with doxorubicin (6 mg kg$^{-1}$), or by intraperitoneal (i.p.) injection of trimelamol (50 mg kg$^{-1}$). For trimelamol, the i.p. route of injection was preferred over the i.v. route because dimethylsulphoxide (DMSO) was required as the solvent. These doses of cytotoxic drugs were the lowest required to cause repeated and reproducible vomiting. In the experiments with dogs, apomorphine (0.1 mg kg$^{-1}$) was injected subcutaneously.

To evoke emesis by X-irradiation, ferrets were closely confined in a ventilated box constructed of perspex 1 mm thick. X-rays were derived from the tungsten anode of a Machlett Model OEG-50 X-ray supply, operating at 50 kV and 20 mA through a berylium window with a 0.18 mm aluminium filter and placed about 25 cm above the ferret. This low energy X-ray beam was just sufficient to cause reproducible emesis; exposure time was 10.4 min.

*Observations* Ferrets given cisplatin or cyclophosphamide and doxorubicin were observed for the onset of emesis (latency period) and the number of emetic episodes over 240 min after injection. Emesis had usually ceased in control animals within this period. The observation period following trimelamol was 210 min, minimising any discomfort caused by the i.p. injection of the large volume of trimelamol and solvent. For dogs given apomorphine, the observation period was 30 min. The observation period following X-irradiation was 120 min, since in control animals, emesis had ceased within this time. Untreated ferrets were observed for 240 min.

In some experiments, additional behavioural events were also monitored, these being subjectively considered to indicate 'nausea'. These events were retching, drinking, defecation, posturing to defecate, urgent tunnelling under the wood shavings in the pen (burrowing), obtrusive licking (salivation?), and urgent backing movements (backing). Behaviours such as playing, foraging, casual burrowing, sleeping, or occasional urgent grooming were observed in control ferrets and were therefore not recorded. All experiments were

Correspondence: G.J. Sanger.
Received 8 February 1988; and in revised form, 3 June 1988.

performed between 0800 and 1400 h, with the same two observers throughout.

*Drugs* Cisplatin (Neoplatin for injection; Bristol Myers), cyclophosamide (Endoxana for injection; WB Pharmaceuticals) and doxorubicin (Adriamycin for injection; Farmitalia) were diluted in water for injection, BP. Trimelamol ($N^2$,$N^4$,$N^6$-trihydroxymethyl-$N^2$,$N^4$,$N^6$-trimethylmelamine; kindly provided by Dr. I. Judson, Institute of Cancer Research, London, UK) was dissolved in DMSO: 5% dextrose [1:10]. Apomorphine hydrochloride was dissolved in 0.05% w/v sodium metabisulphite solution. BRL 43694 (*endo* - N - (9 - methyl - 9 - azabiocyclo[3.3.1]non - 3 - yl) - 1 - methyl - 1H - indazole - 3 - carboxamide hydrochloride; Beecham Pharmaceuticals) was dissolved in water for injection BP or 0.9% saline. Doses of BRL 43694 are given as mg kg$^{-1}$ free base.

*Statistical analysis* Results are given as means ± s.e.m. and were analysed using Student's *t* test. In the Figures, the standard error bars have been omitted for clarity.

### Results

The number of ferrets exposed to emetic stimuli in the absence of anti-emetic therapy was minimised by testing at least one ferret, chosen at random, from each delivery of animals. These ferrets provided the accumulated control data described below.

### *Emesis evoked by cisplatin*

The mean number of emetic episodes for each 10 min period over the 240 min following injection of cisplatin is shown in Figure 1. When BRL 43694 was given in divided dose, 0.5 mg kg$^{-1}$ i.v., 30 min before and 45 min after cisplatin, emesis was prevented (Figure 1; Table I). BRL 43694 gave a dose-related protection against cisplatin-induced emesis down to a dose of 2 × 0.005 mg kg$^{-1}$ i.v. (Table I). Even at this lowest dose there was a highly significant delay in the average onset of emesis ($P<0.001$), with 2 of the 4 ferrets being completely protected.

A single injection of BRL 43694 given 15 min before cisplatin, also dose-dependently reduced the number of emetic episodes (Table I). There was complete protection with 0.5 mg kg$^{-1}$ i.v., and significantly less vomiting than in unprotected ferrets at 0.05 mg kg$^{-1}$ i.v. In the experiments

**Figure 1** Cisplatin-induced emesis in the ferret. (a) Emetic episodes (mean values) shown in 10 min intervals over 240 min after cisplatin (10 mg kg$^{-1}$ i.v.) at time zero [arrow], with saline given at −30 and +45 min as shown (△); ($n=8$). (b) Protection from emesis by BRL 43694 (2 × 0.5 mg kg$^{-1}$ i.v.) given at −30 and at +45 min as shown (▲); ($n=4$). (c) Abolition of vomiting by BRL 43694 (0.5 mg kg$^{-1}$ i.v.) given during emesis (▼); ($n=4$).

with BRL 43694 0.5 mg kg$^{-1}$ i.v., behavioural changes were also monitored and compared with the behaviour of untreated ferrets (Figure 2). Cisplatin-evoked emesis was

**Table I** Cisplatin-induced vomiting in the ferret

| *Emetic stimulus (i.v.)* | *Anti-emetic treatment (mg kg$^{-1}$ i.v.)* | *Latency period to first vomit (min)* | *Number of emetic episodes (over 240 min)* |
|---|---|---|---|
| (a) Divided dose of BRL 43694 | | | |
| Cisplatin | 2 × Saline | 71.0 ± 2.8 ($n=8$) | 16.3 ± 1.7 |
| Cisplatin | BRL 43694 0.5/0.5 | No vomits ($n=4$) | 0[b] |
| | BRL 43694 0.05/0.05 | 230.5 ± 9.5[b] ($n=4$) | 0.5 ± 0.5[b] |
| | BRL 43694 0.005/0.005 | 183.8 ± 32.6[b] ($n=4$) | 1.0 ± 0.6[a] |
| (b) Single dose of BRL 43694 | | | |
| Cisplatin | BRL 43694 0.5 | No vomits ($n=4$) | 0[b] |
| | BRL 43694 0.05 | 141.0 ± 50.1 ($n=3$) | 3.7 ± 1.9[a] |

Ferrets were given cisplatin 10 mg kg$^{-1}$ i.v. BRL 43694 was given (a) 30 min before and 45 min after cisplatin or (b) 15 min before cisplatin. Compared with controls, [a]$P<0.01$; [b]$P<0.001$. If a ferret did not vomit, latency period was taken as equal to the observation period (240 min). Results are given as means ± s.e.m.

**Figure 2** Selected patterns of behaviour of two untreated ferrets monitored over 240 min, with each event being represented by a vertical line. Similar patterns of behaviour were observed in two further ferrets (female) monitored over the same period (results not shown). (defec.) denotes posture to defecate.

accompanied or preceded by prolonged bouts of retching; backing and less frequently, burrowing, accompanied these events. Most of the animals drank occasionally. Three of the four cisplatin-treated animals which received BRL 43694, showed near-normal behaviour (Figure 3), with drinking being the main significant event. The fourth animal had a cluster of burrowing and retching towards the end of the observation period (180–190 min) but this was not accompanied by emesis.

Once cisplatin-induced emesis was established (90 min after injection), a single dose of BRL 43694 (0.5 mg kg$^{-1}$ i.v.), given during an emetic episode, abolished further emesis usually within 30 s after injection (Figure 1). Thereafter, the ferrets were protected from further emesis.

*Emesis evoked by cyclophosphamide and doxorubicin*

The mean number of emetic episodes for each 10 min period following injection of cyclophosphamide and doxorubicin is shown in Figure 4. BRL 43694 given in divided dose, 0.5 mg kg$^{-1}$ i.v. 30 min before and 30 min after the cytotoxic drugs, completely prevented emesis in one ferret, and greatly reduced emesis (1, 1 and 2 episodes respectively) in the other 3 ferrets (Figure 4). Behavioural changes associated with emesis were not measured in these experiments.

Once emesis was established, a single dose of BRL 43694 (0.5 mg kg$^{-1}$ i.v.) given during an emetic episode (at 50 min or 80 min, following cytotoxic drug injections) abolished emesis, usually within 30 s after injection (Figure 4). Thereafter, the ferrets were protected from further emesis.

*Emesis evoked by trimelamol*

There was considerable variation in the emetic response to trimelamol, with the onset of emesis ranging from 6 min to 80 min after injection; the mean time of onset of emesis is shown in Table II. Two control ferrets injected with an equivalent volume of 10% DMSO in 5% dextrose did not vomit (Table II) and appeared unaffected by this treatment. BRL 43694 prevented emesis (Table II) and associated behavioural disturbances (Figure 5) when given in divided dose, 0.5 mg kg$^{-1}$ i.v. 30 min before and 45 min after trimelamol.

*Emesis evoked by X-irradiation*

There was a rapid and uniform response to X-irradiation in control animals, with emesis usually occurring within 20 min of the start of exposure and remaining severe for ~1 h (Figure 6). BRL 43694 (0.5 mg kg$^{-1}$i.v.) given 5 min before exposure, prevented vomiting in all 4 ferrets tested (Figure 6). Compared with untreated ferrets, the behavioural changes associated with emesis were also prevented by this dose of BRL 43694 (Figure 7).

BRL 43694 (0.5 mg kg$^{-1}$ i.v.) given to ferrets during the peak emetic period (~20 min after the removal from the X-ray source), prevented subsequent emesis (Figure 6) and associated behavioural changes (Figure 8).

Oral administration of BRL 43694 (0.5 mg kg$^{-1}$) 60 min before irradiation abolished emesis in 2 of 3 ferrets, and considerably delayed its onset in the third (a mean value is shown in Table III). There was still some delay in onset at a tenth of this dose of BRL 43694 (0.05 mg kg$^{-1}$), as well as a reduction in the number of emetic episodes (Table III).

*Emesis evoked by apomorphine*

BRL 43694 0.1 mg kg$^{-1}$ i.v. given 15 min before apomor-

**Figure 3** Selected patterns of behaviour evoked by cisplatin (10 mg kg$^{-1}$ i.v.) in control ferrets ($n$=8) and in treated ferrets given BRL 43694 (0.5 mg kg$^{-1}$ i.v.; $n$=4) 15 min before cisplatin. The behavioural events (each represented by a vertical line) were monitored over 240 min after dosing with cisplatin (10 mg kg$^{-1}$ i.v.) at time zero. (defec.) denotes posture to defecate.

**Figure 4** Emesis induced by cyclophosphamide and doxorubicin in the ferret. (a) Emetic episodes (mean values) shown in 10 min intervals over 240 min after cyclophosphamide (80 mg $kg^{-1}$ i.v.) and doxorubicin (6 mg $kg^{-1}$ i.v.) given at time zero (arrow), with saline given at −30 and +30 min as shown (△); ($n$=7). (b) Protection from emesis by BRL 43694 (2×0.5 mg $kg^{-1}$ i.v.) given at −30 and +30 min as shown (▲); ($n$=4). (c) Abolition of vomiting in 2 ferrets by BRL 43694 (0.5 mg $kg^{-1}$ i.v.) given during emesis as shown (▼); ($n$=4).

phine did not protect the dogs against the subsequent emesis. The number of vomits evoked by apomorphine in dogs injected with saline (9.5±2.5 vomits) was not significantly different when BRL 43694 was injected (9.3±2.9 vomits; $n$=4).

### Discussion

Our results show that BRL 43694 is a highly potent and efficacious anti-emetic in the ferret, whether the stimulus is one of a number of severely emetogenic cytotoxic drugs or exposure to X-irradiation. We have previously demonstrated that results obtained with other anti-emetic drugs in ferrets can correlate with their anti-emetic potential in patients (Miner *et al.*, 1987). Our present work with BRL 43694 therefore suggests that this compound may provide considerable relief for cancer patients undergoing therapy. Furthermore, they provide yet more evidence in support of our original proposal for a crucial involvement of 5-$HT_3$ receptors in the mechanisms of severe emesis (Miner *et al.*, 1986; Miner & Sanger, 1986).

In contrast to similar experiments in ferrets, using high doses of metoclopramide (Miner *et al.*, 1987), in which emesis was simply reduced, BRL 43694 prevented emesis when given either prophylactically (i.v. or p.o.) or after emesis had begun. In the latter experiments, a single dose of BRL 43694, given at a peak emetic period and during emesis itself, dramatically abolished vomiting within seconds on every occasion of testing. BRL 43694 may, therefore, have considerable flexibility within the clinic, abolishing emesis whenever it is used.

During the course of monitoring emetic episodes, it became obvious that a record of emesis alone undervalued the experiments. Emesis was always accompanied by marked disturbances in behaviour, and this has been demonstrated previously for tetralin-evoked emesis in marmosets (Costall *et al.*, 1986*a*). In the present experiments with ferrets, retching could occur in the absence of vomiting (or vice versa), and was frequently preceded or accompanied by urgent burrowing and backing, with which it was clearly associated. Initially it was thought that frequent defaecation or posturing to defaecate might be an index of general intestinal disturbance and discomfort in unprotected ferrets receiving the emetic stimuli, but this could not be demonstrated here; likewise tongue protrusion or licking were monitored as a reflection of the salivation experienced in human nausea, but again, they did not accompany emesis in these experiments. In general, the different emetic stimuli evoked behavioural disturbances in waves, usually culminating in emesis, and reminiscent of the human condition of nausea. Whether these findings equate with human nausea or not, for the ferret they clearly portray considerable restlessness and discomfort closely associated with emesis. That this can be prevented, by either prophylactic or intervention treatment with BRL 43694, implies that 5-$HT_3$ receptors may also be involved in the nausea associated with aggressive anti-cancer therapies in the clinic.

We have previously shown that 5-$HT_3$ receptors may be involved in the mechanisms of emesis evoked by cisplatin (Miner & Sanger, 1986; Miner *et al.*, 1986, 1987), cyclophosphamide (Andrews *et al.*, 1987*b*), cyclophosphamide plus doxorubicin (Miner *et al.*, 1987) or by total body X-irradiation (Andrews *et al.*, 1987; Miner *et al.*, 1987). Similar conclusions have also been reached by others in ferrets (Costall *et al.*, 1986*b*; 1987; Andrews *et al.*, 1987*a*; Hawthorn *et al.*, 1988) and in cancer patients receiving cisplatin (Leibundgut & Lancranjan, 1987; Carmichael *et al.*, 1988) and non-cisplatin cytotoxic therapy (Cunningham *et al.*,

**Table II** Trimelamol-induced vomiting in the ferret

| *Emetic stimulus (i.p.)* | *Anti-emetic treatment (mg $kg^{-1}$ i.v.)* | *Latency period to first vomit (min)* | *Number of emetic episodes (over 210 min)* |
|---|---|---|---|
| DMSO/dextrose + trimelamol | None | 35.8±16 ($n$=4) | 12.3±2.8 |
| DMSO/dextrose + trimelamol | BRL 43694 0.5/0.5 | No vomits ($n$=3) | $0^b$ |
| DMSO/dextrose | None | No vomits ($n$=2) | $0^b$ |

Ferrets were given trimelamol 50 mg $kg^{-1}$ i.p. BRL 43694 (0.5 mg $kg^{-1}$) was given 30 min before and 45 min after trimelamol. Compared with controls $^aP<0.05$, $^bP<0.001$. Results are given as means±s.e.m.

**Figure 5** Selected patterns of behaviour evoked by trimelamol ($50\,mg\,kg^{-1}$ i.p.) in control ferrets ($n=4$) and in treated ferrets, given BRL 43694 ($2\times0.5\,mg\,kg^{-1}$ i.v.; $n=3$) 30 min before and 45 min after trimelamol. The behavioural events (each represented by a vertical line) were monitored over 210 min after dosing with trimelamol at time zero. (defec.) denotes posture to defecate.

**Figure 6** Radiation-induced emesis in the ferret. (a) Emetic episodes (mean values) shown in 10 min intervals over 120 min after exposure to X-irradiation from 0 to 10.4 min (arrow); ($n=5$). (b) Protection from emesis by BRL 43694 ($0.5\,mg\,kg^{-1}$ i.v.) given at $-5$ min as shown (▲); ($n=4$). (c) Abolition of vomiting by BRL 43694 ($0.5\,mg\,kg^{-1}$ i.v.) given immediately after an emetic episode had occurred (▼); ($n=4$).

1987). We now show that $5\text{-}HT_3$ receptors may also be involved in the mechanisms of emesis evoked by trimelamol, a potentially useful anti-cancer drug (see Rutty *et al.*, 1986). In cancer patients, the emesis caused by trimelamol can be severe and dose-limiting, discouraging a more widespread use. The advantages of concurrent use of BRL 43694 with severely emotogenic anti-cancer drugs are therefore obvious, illustrating how the current use of such drugs might change with the advent of very effective antiemetic agents.

BRL 43694 is a selective $5\text{-}HT_3$ receptor antagonist with little or no affinity for a wide range of receptors other than the $5\text{-}HT_3$ receptor (Fake *et al.*, 1987). In particular, BRL 43694 does not antagonise dopamine $D_2$ receptors, and therefore should be free of the extrapyramidal side-effects associated with high intravenous doses of metoclopramide. Consistent with the poor affinity for $D_2$ receptors is the failure of BRL 43694 to antagonise apomorphine-evoked emesis in dogs. Although we have not demonstrated a similar lack of activity in ferrets, these experiments do suggest that BRL 43694 may not suppress the entire emetic reflex, but more specifically block those stimuli that evoke emesis by acting through a $5\text{-}HT_3$ receptor-mediated mechanism. It is thought that these $5\text{-}HT_3$ receptors are located on visceral afferent nerve terminals within the abdomen and also at an 'extra-abdominal' site (Andrews & Hawthorn, 1987). The latter $5\text{-}HT_3$ receptor site is not yet precisely defined, but may be located within the area postrema, containing the emetic chemoreceptor trigger zone. Thus, the ability of drugs or radiation to cause emesis, and their

**Figure 7** Selected patterns of behaviour evoked by X-irradiation in control ferrets ($n=4$) and in treated ferrets, given BRL 43694 ($0.5\,mg\,kg^{-1}$ i.v.; $n=2$) 15 min before X-irradiation. The behavioural events (each represented by a vertical line) were monitored over a 120 min observation period following X-irradiation (10.4 min exposure; shaded band). (defec.) denotes posture to defecate.

**Figure 8** Patterns of behaviour in ferrets given a single dose of BRL 43694 ($0.5\,mg\,kg^{-1}$ i.v.; $n=2$) immediately after a group of emetic episodes and during the peak emetic period following X-irradiation (arrow). The behavioural events (each represented by a vertical line) were monitored over 120 min from the start of X-irradiation (10.4 min; shaded band). (defec.) denotes posture to defecate.

respective latency periods, could depend on their potential to increase the release and/or synthesis of 5-HT. For example, in small laboratory animals (which do not vomit), total body X-irradiation may cause a sudden release of 5-HT from the gut, depleting the mucosal enterochromaffin cells of the 5-HT which they normally contain (Matsouka *et al.*, 1962). The released 5-HT could therefore activate gastrointestinal and/or hepatic primary afferent fibres, which eventually synapse within the area postrema. Whole body or head irradiation of rats may also increase 5-HT synthesis within the lower brain stem (Altman *et al.*, 1970). A sustained increase of 5-HT synthesis within the brainstem might therefore be expected to cause emesis by a more direct action at the area postrema.

For cytotoxic drugs, there has been little or no previous association with 5-HT. Gunning *et al.* (1987) described an increase in 5-HT and 5-hydroxyindoleacetic acid (5-HIAA) extracted from the small intestinal mucosa of ferrets previously injected with cisplatin ($9\,mg\,kg^{-1}$ i.p. for 2 days); there was no change in the amount of 5-HT extracted from the gastric mucosa or from the hypothalamus. Furthermore, cisplatin was shown to cause selective damage of the small intestine, but not the stomach. However, although these results support the involvement of 5-HT in the mechanisms of emesis, the measurements of 5-HT and 5-HIAA were made after emesis should have occurred. Since mechanical stimuli can release 5-HT from the gut (Bennett *et al.*, 1966), these particular changes in 5-HT release cannot, therefore, necessarily be taken as evidence for an association between 5-HT and the emetic potential of cisplatin.

In contrast to metoclopramide, interdigestive gastric motility is not normally stimulated by BRL 43694 (Fake *et al.*, 1987). The ability of metoclopramide to stimulate gastrointestinal motility may provoke diarrhoea in up to 25% of cancer patients receiving the drug in combination with

**Table III** Radiation-induced vomiting in the ferret

| *Emetic stimulus* | *Anti-emetic treatment ($mg\,kg^{-1}$ p.o.)* | *Latency period to first vomit (min)* | *Number of emetic episodes (over 120 min)* |
|---|---|---|---|
| X-irradiation | Saline | $21.0 \pm 0.8$ ($n=7$) | $26.0 \pm 3.5$ |
| X-irradiation | BRL 43694 0.5 | $104.7 \pm 15.3^{b}$ ($n=3$) | $2.3 \pm 2.3^{a}$ |
| | BRL 43694 0.05 | $51.8 \pm 6.3^{b}$ ($n=4$) | $6.3 \pm 2.1^{a}$ |

Ferrets were X-irradiated for 10.4 min. BRL 43694 was given orally 60 min before the start of irradiation. Compared with controls, $^{a}P<0.01$; $^{b}P<0.001$. If a ferret did not vomit, latency period was taken as equal to the observation period (120 min). Results are given as means $\pm$ s.e.m.

cytotoxic agents (Strum *et al.*, 1982). Selective 5-$HT_3$ receptor antagonists such as BRL 43694 may therefore be expected to have minimal problems associated with diarrhoea and abdominal cramps.

In conclusion, our experiments with BRL 43694 in ferrets demonstrate the remarkable anti-emetic potential of this compound. Furthermore, since BRL 43694 can also prevent the behavioural changes which are associated with emesis in ferrets, both nausea and emesis may be prevented by this compound. BRL 43694 is therefore currently undergoing clinical trials in cancer patients. Early results with BRL 43694 show a promising anti-emetic and anti-nauseant activity against cisplatin-containing therapies, when dosed prophylactically (Carmichael *et al.*, 1988; Cassidy *et al.*, 1988; Gumbell *et al.*, 1988) or after emesis has begun (Cassidy *et al.*, 1988). BRL 43694 did not cause extrapyramidal symptoms and was free of the sedation and other side effects detected with dopamine receptor antagonists, benzodiazepine or corticosteroid treatments. It is therefore hoped that the use of this drug will allow effective management of the debilitating nausea and vomiting associated with aggressive anti-cancer therapy.

We thank Mr R. Collie and Mrs V. Smith for assistance with the ferret cannulations and Mr. G. Heald for his radiation expertise. We are also grateful to Dr G. Rapeport and Dr I. Judson for suggesting the studies with trimelamol.

### References

ALTMAN, K.I., GERBER, G.B. & OKADA, S. (1970). Radiation biochemistry. In *Tissues and Body Fluids*. Vol. II, p. 307. Academic Press.

ANDREWS, P.L.R. & HAWTHORN, J. (1987). Evidence for an extra-abdominal site of action for the 5-$HT_3$ receptor antagonist BRL 24924 in the inhibition of radiation-evoked emesis in the ferret. *Neuropharmacol.*, **26**, 1367.

ANDREWS, P.L.R. & BAILEY, H.E., HAWTHORN, J., STABLES, R. & TYERS, M.B. (1987*a*). GR 38032F, a novel 5-$HT_3$ receptor antagonist, can abolish emesis induced by cyclophosphamide or radiation in the ferret. *Br. J. Pharmacol. Proc. Suppl.*, **91**, 417P (*abstract*).

ANDREWS, P. L. R., HAWTHORN, J. & SANGER, G.J. (1987*b*). The effect of abdominal visceral nerve lesions and a novel 5-HT M-receptor antagonist on cytotoxic and radiation-evoked emesis in the ferret. *J. Physiol.*, **382**, 47P (*abstract*).

BENNETT, A., BUCKNELL, A. & DEAN, A.C.B. (1966). The release of 5-hydroxytryptamine from the rat stomach *in vitro*. *J. Physiol.*, **182**, 57.

BOYLE, E.A., MINER, W.D. & SANGER, G.J. (1987*a*). Anti-emetic activity of BRL 43694, a novel 5-$HT_3$ receptor antagonist. *Br. J. Cancer*, **56**, 227.

BOYLE, E.A., MINER, W.D. & SANGER, G.J. (1987*b*). Different anti-cancer therapies evoke emesis by mechanisms that could be blocked by the 5-$HT_3$ receptor antagonist, BRL 43694. *Br. J. Pharmacol., Proc. Suppl.*, **91**, 418P (*abstract*).

CARMICHAEL, J., CANTWELL, B.M.J., EDWARDS, C.M., RAPEPORT, W.G. & HARRIS, A.L. (1988). The serotonin type 3 receptor antagonist BRL 43694 and nausea and vomiting induced by cisplatin. *Br. Med. J.*, **297**, 110.

CASSIDY, J., LEWIS, C., RAPEPORT, W.G. & 4 others (1988). Anti-emetic activity of BRL 43694, a selective 5-$HT_3$ receptor antagonist, in cancer chemotherapy. *Br. J. Cancer*, **58**, 275 (*abstract*).

COSTALL, B., DOMENEY, A.M. & NAYLOR, R.J. (1986*a*). A model of nausea and emesis in the common marmoset. *Br. J. Pharmacol., Proc. Suppl.*, **88**, 375P (*abstract*).

COSTALL, B., DOMENEY, A.M., NAYLOR, R.J. & TATTERSALL, F.D. (1986*b*). 5-Hydroxytryptamine M-receptor antagonism to prevent cisplatin-induced emesis. *Neuropharmacol.*, **25**, 959.

COSTALL, B., DOMENEY, A.M., GUNNING, S.J., NAYLOR, R.J., TATTERSALL, F.D. & TYERS, M.B. (1987). GR 38032F: A potent and novel inhibitor of cisplatin-induced emesis in the ferret. *Br. J. Pharmacol., Proc. Suppl.*, **90**, 90P (*abstract*).

CUNNINGHAM, D., HAWTHORN, J., POPLE, A. & 4 others (1987). Prevention of emesis in patients receiving cytotoxic drugs by GR 38032F, a selective 5-$HT_3$ receptor antagonist. *Lancet*, **i**, 1461.

FAKE, C.S., KING, F.D. & SANGER, G.J. (1987). BRL 43694: A potent and novel 5-$HT_3$ receptor antagonist. *Br. J. Pharmacol., Proc. Suppl.*, **91**, 335P (*abstract*).

FLORCZYK, A.P. & SCHURIG, J.E. (1981). A technique for chronic jugular catherization in the ferret. *Pharmacol. Biochem. Behav.*, **14**, 255.

GUMBELL, L.A., QUALMANN, C., PERREN, T., FORGESON, G., CALVERT, A.H., THOMPSON, S. & RAPEPORT, W.G. (1988). An open pilot study of the 5-$HT_3$ receptor antagonist, BRL 43694, an effective antiemetic in refractory highly emetogenic cytotoxic drug-induced emesis. *Br. J. Cancer*, **58**, 274 (*abstract*).

GUNNING, S.J., HAGAN, R.M. & TYERS, M.B. (1987). Cisplatin induces biochemical and histological changes in the small intestine of the ferret. *Br. J. Pharmacol., Proc. Suppl.*, **90**, 135P (*abstract*).

HAWTHORN, J., OSTLER, K.J. & ANDREWS, P.L.R. (1988). The role of the abdominal visceral innervation and 5-hydroxytryptamine M-receptors in vomiting induced by the cytotoxic drugs cyclophosphamide and cisplatin in the ferret. *Quart. J. Exp. Physiol.*, **73**, 7.

LEIBUNDGUT, U. & LANCRANJAN, I. (1987). First results with ICS 205-930 (5-$HT_3$ receptor antagonist) in prevention of chemotherapy-induced emesis. *Lancet*, **i**, 1198.

MATSOUKA, O., TSUCHIYA, T. & FURUKAWA, Y. (1962). The effect of X-irradiation on 5-hydroxytryptamine (serotonin) contents in the small intestines of experimental animals. *J. Radiat. Res.*, **302**, 104.

MINER, W.D. & SANGER, G.J. (1986). Inhibition of cisplatin-induced vomiting by selective 5-hydroxytryptamine M-receptor antagonism. *Br. J. Pharmacol.*, **88**, 497.

MINER, W.D., SANGER, G.J. & TURNER, D.H. (1986). Comparison of the effect of BRL 24924, metoclopramide and domperidone on cisplatin-induced emesis in the ferret. *Br. J. Pharmacol., Proc. Suppl.*, **88**, 374P. (*abstract*).

MINER, W.D., SANGER, G.J. & TURNER, D.H. (1987). Evidence that 5-hydroxytryptamine$_3$ receptors mediate cytotoxic drug and radiation-evoked emesis. *Br. J. Cancer*, **56**, 159.

RUTTY, C.J., JUDSON, I.R., ABEL, G., GODDARD, P.M., NEWELL, D.R. & HARRAP, K.R. (1986). Preclinical toxicology, pharmacokinetics and formulation of $N^2$,$N^4$,$N^6$-trihydroxymethyl-$N^2$,$N^4$,$N^6$-trimethylmelamine (Trimelamol), a water-soluble cytotoxic S-triazine which does not require metabolic activation. *Cancer Chemother. Pharmacol.*, **17**, 251.

STRUM, S.B., McDERMED, J.E., OPFELL, R.W. & RIECH, L.P. (1982). Intravenous metoclopramide: An effective antiemetic in cancer chemotherapy. *J. Am. Med. Assoc.*, **274**, 2683.